# Molecular Mechanisms of Methamphetamine-Induced Addiction via TAAR1 Activation

**DOI:** 10.1021/acs.jmedchem.4c01961

**Published:** 2024-10-02

**Authors:** Yun Lin, Jiening Wang, Fan Shi, Linlin Yang, Shan Wu, Anna Qiao, Sheng Ye

**Affiliations:** † Tianjin Key Laboratory of Function and Application of Biological Macromolecular Structures, School of Life Sciences, Tianjin University, 92 Weijin Road, Nankai District, Tianjin 300072, China; ‡ State Key Laboratory of Biocatalysis and Enzyme Engineering, Hubei Collaborative Innovation Center for Green Transformation of Bio-Resources, Hubei Key Laboratory of Industrial Biotechnology, School of Life Sciences, Hubei University, Wuhan, Hubei 430062, China; § Department of Pharmacology, School of Basic Medical Sciences, Zhengzhou University, Zhengzhou 450001, China

## Abstract

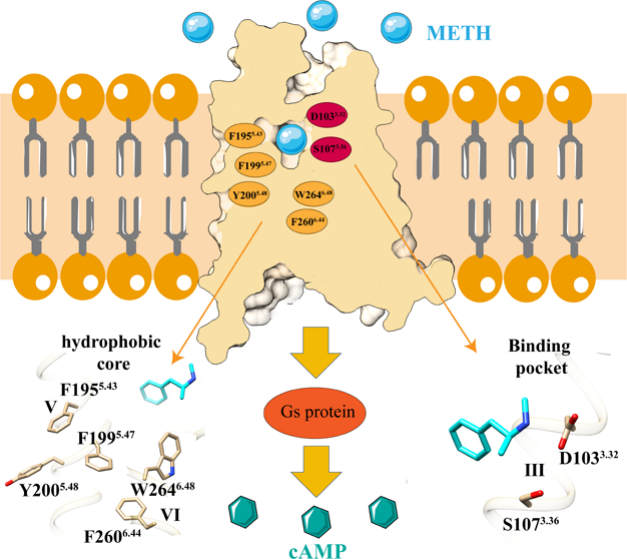

Trace amine-associated receptor 1 (TAAR1), a member of the trace amine receptor family, recognizes various trace amines in the brain, including endogenous β-phenylethylamine (PEA) and methamphetamine (METH). TAAR1 is a novel target for several neurological disorders, including schizophrenia, depression, and substance abuse. Herein, we report the structure of the human TAAR1–G_s_ protein complex bound to METH. Using functional studies, we reveal the molecular basis of METH recognition by TAAR1, and potential mechanisms underlying the selectivity of TAAR1 for different ligands. Molecular dynamics simulations further elucidated possible mechanisms for the binding of chiral amphetamine (AMPH)-like psychoactive drugs to TAAR1. Additionally, we discovered a hydrophobic core on the transmembrane helices (TM), TM5 and TM6, explaining the unique mechanism of TAAR1 activation. These findings reveal the ligand recognition pattern and activation mechanism of TAAR1, which has important implications for the development of next-generation treatments for substance abuse and various neurological disorders.

## Introduction

Since the 21st century, amphetamine (AMPH)-like drugs have become more prevalent, particularly methamphetamine (METH). METH is a potent central nervous system (CNS) stimulant discovered in 1893.^1^ It was once used to treat attention-deficit hyperactivity disorder (ADHD) and obesity but is now commonly abused recreationally.^2^ At low doses, METH can improve mood; increase alertness, attention, and energy in fatigued individuals; reduce appetite; and promote weight loss.^3−5^ However, at very high doses, it can cause psychosis, skeletal muscle breakdown, seizures, and brain hemorrhage.^6^ Long-term high-dose use can cause unpredictable rapid mood swings, stimulant psychosis (i.e., delusions, hallucinations, delirium, and paranoia), and violent behavior.^7,8^ Recreationally, METH is reported to improve mood, increase energy, and boost libido, enabling users to engage in prolonged sexual activity while using the drug.^9^ METH is known for its high addiction potential (long-term or high-dose use is likely to result in compulsive drug use) and high dependency potential (cessation is expected to cause withdrawal symptoms).^10,11^ Withdrawal after extensive METH use can lead to acute withdrawal syndrome, which can endure for months, exceeding typical withdrawal periods of several weeks.^10,12^ Drug addiction induced by AMPH-like drugs is a chronic relapsing brain disorder characterized by drug seeking, abuse, and harmful consequences. In humans, METH is neurotoxic to midbrain dopaminergic neurons and, at high doses, serotonergic neurons. This form of addiction alters brain circuits, impairing the reward system and causing functional consequences in stress management and self-control.^13−15^

Owing to the substantial harm it causes, which is comparable to that of traditional narcotics, METH is listed in Schedule II of the United Nations Convention on Psychotropic Substances. Many countries restrict or prohibit the production, distribution, sale, and possession of METH. However, in February 2023, the Australian government approved 3,4-methylenedioxymethamphetamine (ecstasy/MDMA) for the treatment of post-traumatic stress disorder (PTSD). Furthermore, there is a trend in the United States toward relaxing regulations on psychotropic drugs.^16^ Consequently, understanding the addiction mechanisms of AMPH-type drugs and developing treatments for addiction are urgently needed.

**Figure 1:**
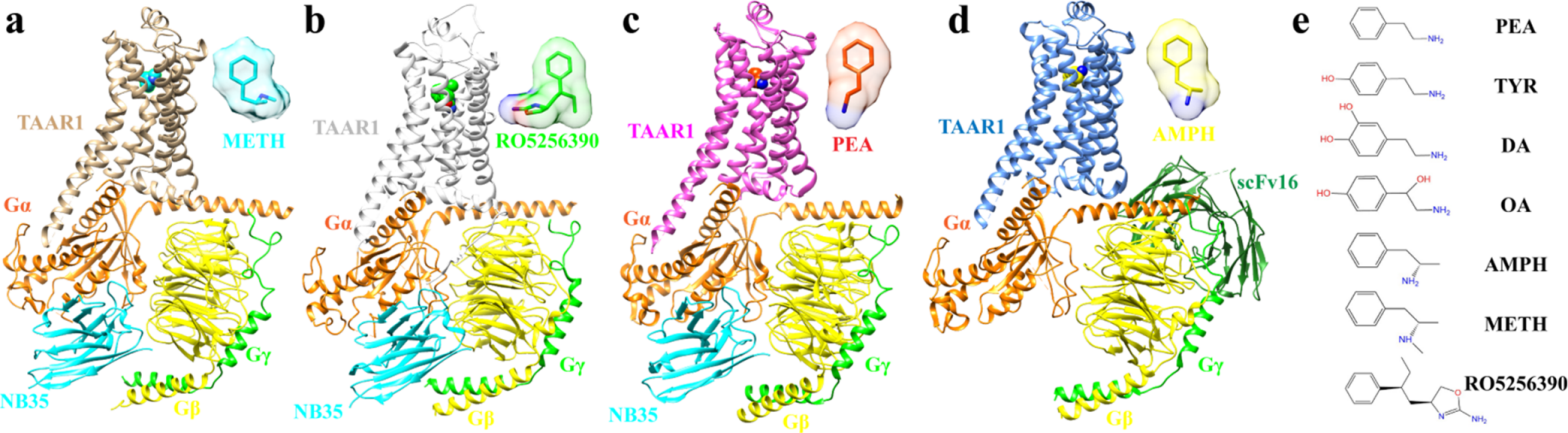
Overall cryo-EM structures of the METH–TAAR1–G_s_ complexes. (a–d) Structures of the TAAR1–G_s_ with METH (PDB ID: 9JKQ) and RO5256390 (PDB ID: 8UHB)/PEA (PDB ID: 8W89)/AMPH (PDB ID: 8JSO). The receptors are colored ivory (a), light gray (b), pink (c), and cornflower blue (d), and the ligands are colored cyan (a), green (b), orange-red (c), and yellow (d), respectively. Gα_s_, Gβ, Gγ, Nb35, and scFv16 are colored orange, yellow, green, cyan, and forest green, respectively. (e) Chemical structures of phenylethylamine (PEA), tyramine (TYR), dopamine (DA), octopamine (OA), amphetamine (AMPH), methamphetamine (METH), and RO5256390.

Early literature reported that trace amine-associated receptor 1 (TAAR1) is activated by AMPH-like drugs, including AMPH, METH, and 3,4-methylenedioxyamphetamine (MDA). TAAR1 plays a key role in regulating addiction responses induced by these drugs.^17−20^ As a G protein-coupled receptor (GPCR), TAAR1 is distributed both on the cell membrane and intracellularly, with a predominant intracellular localization.^21^ This is in contrast to the dopamine (DA) receptor D1R, which is primarily located on the cell membrane.^22^ Most TAAR1 ligands, including tyramine (TYR), phenylethylamine (PEA), octopamine (OA), DA, etc. (Figure 1e), are synthesized in the cytoplasm of monoaminergic cells. These monoamines, along with exogenous AMPH-like drugs, enter cells via monoamine transporters. Therefore, TAAR1 primarily exerts its function by binding agonists in intracellular environments. TAAR1 mainly couples to heterotrimeric G protein, G_s_, to stimulate cyclic adenosine monophosphate (cAMP) production.^22,23^ TAAR1 activation by trace amines modulates neurotransmission in DA, glutamine, and serotonin neurons in the CNS.^24−30^ Notably, PEA shares a phenylethylamine moiety with the psychotropic drugs (Figure 1e) AMPH and its derivative METH, leading to the hypothesis that PEA is an endogenous AMPH.^31,32^ Low PEA levels have been associated with depression,^33^ whereas elevated PEA levels have been linked to schizophrenia, mania symptoms, and the antidepressant effects of exercise.^32,34,35^

TAAR1 Dysfunction is implicated in psychiatric disorders such as schizophrenia, depression, and addiction.^36−38^ TAAR1 activation has been found to attenuate psychostimulant-associated abuse behavior, whereas knockout of TAAR1 potentiates it.^18^ In the last decades, several full and partial TAAR1 agonists have been synthesized.^26,39−44^ For example, the efficacy of full agonist RO5256390 is similar to that of the endogenous TAAR1 agonist PEA, whereas the partial agonist RO5263397 shows lower efficacy. These selective TAAR1 agonists not only improve schizophrenic behavior but also attenuate cocaine, METH, and nicotine addiction.^17,45^ Because TAAR1 plays in mood, behavior, and cognition, agonists that selectively target TAAR1 show promise in treating psychiatric disorders such as schizophrenia, depression, and addiction.^17−19,46−48^ Therefore, understanding the molecular mechanism of binding of METH to TAAR1 is important.

To gain a structural understanding of ligand recognition and signal transduction by TAAR1, we used cryo-electron microscopy (cryo-EM) to identify the structure of the METH-bound TAAR1 and G_s_ complex. Furthermore, we performed pharmacological characterization based on these structural features. These data provide important insights for further elucidating the physiological mechanisms of METH.

### Overall Structures of METH-Bound TAAR1-G_s_ Complex

To obtain the agonist-bound TAAR1-G_s_ complex, an engineered TAAR1 construct was designed by truncating eight amino acids at the C-terminus, fusing the apocytochrome b562 fusion protein BRIL, and introducing two mutations: F112^3.41^W and H63^2.44^V (superscripts indicate nomenclature according to the Ballesteros–Weinstein numbering system). Signaling assays revealed that although these modifications had a minimal effect on the agonistic activity potency (Figure S1a and Table S2), they increased the basal activity of the engineered TAAR1. This indicates that in the absence of agonists, the engineered TAAR1 adopted an active state more readily than the wild type (WT) TAAR1. Each modification increased the basal activity, with F112^3.41^W making the greatest contribution (Figure S1b,c and Table S2). The final engineered construct increased the basal activity by >100% compared to WT TAAR1 (Figure S1b and Table S2). The engineered TAAR1 was coexpressed in insect cells with human Gα_s_, Gβ_1_, and Gγ_2_ as well as G_s_ protein-stabilizing nanobody NB35. The synthesis of METH ligands was, respectively, added to stabilize the nucleotide-free TAAR1-G_s_ complexes. The complexes were purified to homogeneity for single-particle cryo-EM analysis.

The structures of the METH-bound TAAR1-G_s_ complexes were determined using global resolutions of 2.66 Å (Figure 1a). The relatively high-resolution density maps of the complexes allowed us to model most portions of TAAR1 from residues K15 to I323, the entire METH molecule, the G_s_ heterotrimer, and Nb35. The N-terminal region (1–14 amino acids) was weak, which may have been caused by an N-glycosylation motif (N10-X-S12) of TAAR1. Intracellular loop 3 (ICL3) of TAAR1 and the α-helical domain of Gα_s_ were also poorly observed and not modeled because of their flexibility.

To further investigate the mechanism by which METH activates TAAR1, we expanded our research based on the structural analysis of the METH-TAAR1-G_s_ complex. We also reviewed several existing TAAR1 structures, such as the RO5256390-TAAR1-G_s_ complex (PDB ID: 8UHB), the PEA-TAAR1-G_s_ complex (PDB ID: 8W89), and the AMPH-TAAR1-G_s_ complex (PDB ID: 8JSO) (Figure 1b–d). These structures all adopt similar folding patterns and, in terms of the overall complex structures, closely resemble other reported G protein complexes of activated GPCRs,^49^ indicating that they represent the active state of TAAR1. After comparing the METH-TAAR1-G_s_ complex to the other three structures, we found that although the binding pockets they occupied were largely consistent, there were subtle yet important differences. These differences might be the key to the distinct physiological functions exhibited by these ligands.

### Recognition Mode of METH by TAAR1

METH is an AMPH-type psychostimulant with high abuse potential and substantial neuropsychotoxicity.^50^ Importantly, it has been shown that METH-induced DA efflux is dependent on TAAR1 and its downstream signaling, suggesting that TAAR1 is an essential mediator of the actions of METH.^51^

**Figure 2:**
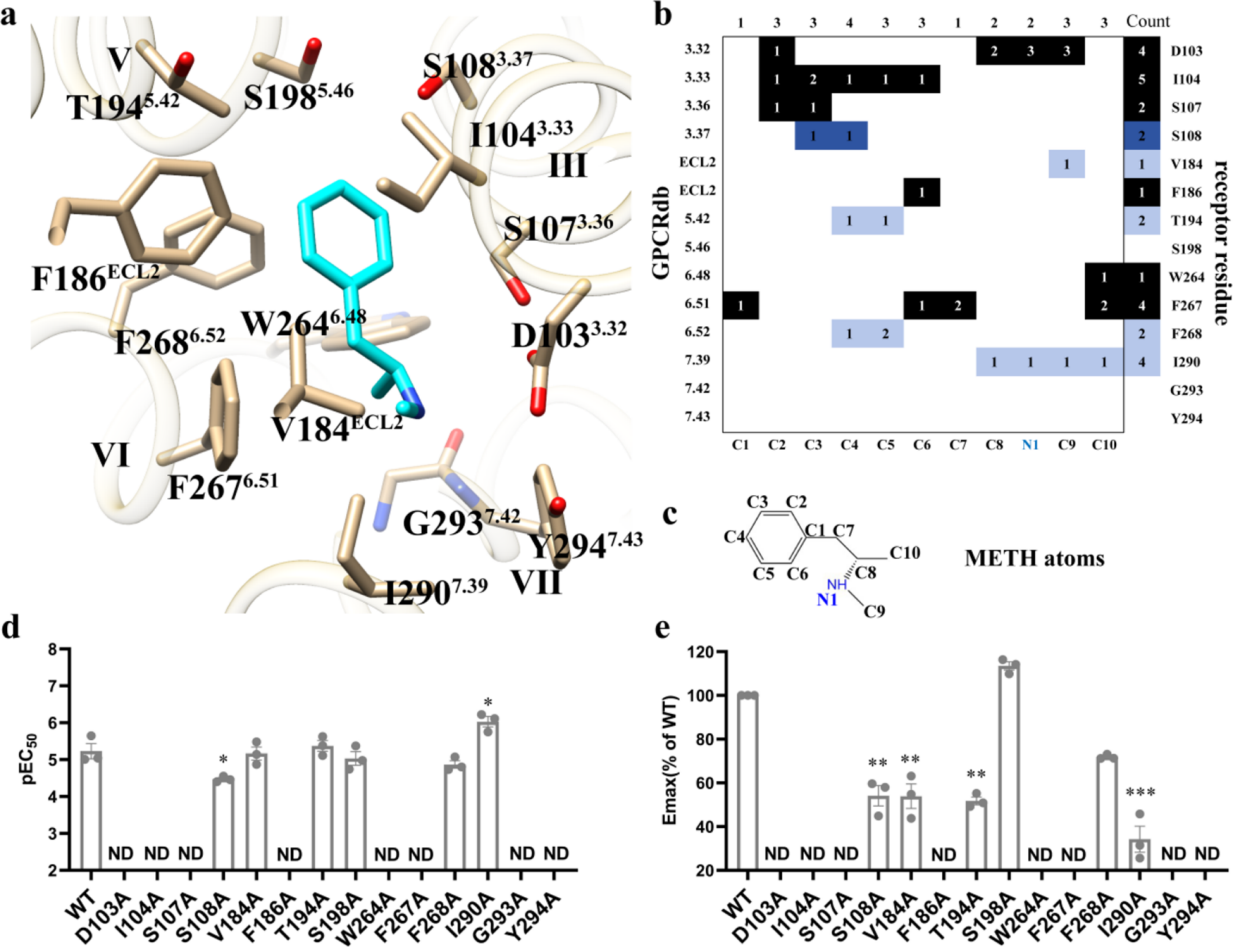
Recognition of METH by TAAR1. (a) Interactions between METH (cyan) with TAAR1. The receptor is colored ivory (PDB ID: 9JKQ). (b) Receptor residue-ligand atom contact plot with the ligand atoms on the *x* axis and the receptor’s GPCRdb and residue numbers on the *y* axis. Two residues are listed as a contact if the distance between the two atoms minus their van der Waals radii equals 0.5 Å or less, corresponding to a maximum distance of ∼4.2 Å. The number of noncovalent contacts between a receptor residue and ligand atom is shown in each square of the heatmap. Box colors in the heatmap refer to the pharmacological effect of the mutation [efficacy affected (light blue), potency affected (ocean blue), both efficacy and potency affected (dark blue), or no measurable signaling (black)]. The number of receptor residues contacted by each ligand atom and the number of ligand atoms contacted by each receptor residue are indicated in boxes at the top and right-hand side of the heatmap, respectively. (c) Chemical structure of METH below the heatmap indicates the labeling of adrenaline atoms used for the *x* axis. (d, e) G_s_-cAMP accumulation results of WT TAAR1 and TAAR1 mutants activated by METH. Activities of METH are identified as pEC_50_ (d) and *E*_max_ (e). *E*_max_ data are normalized to the percentage of the reference agonist METH. Data in (d, e) are mean ± s.e.m of three independent experiments performed in technical triplicate. *P < 0.05, **P < 0.01, ***P < 0.001, (one-way ANOVA followed by Dunnett post test,compared with the response of the WT). ND, not detected. A detailed statistical evaluation is provided in Table S3. Source data are available as a Source Data file.

The cryo-EM map showed the density of METH in the orthosteric binding pocket (Figure 2a). METH comprised a phenylpropyl group and an amino-methyl group. The binding pocket of METH comprised 14 residues of TM3, TM5, TM6, and TM7 and extracellular loop 2 (ECL2) (Figure 2b).

To validate the METH binding mode at the orthosteric site, we performed individual mutations of most of the ligand pockets, assessed their expression levels, and tested the activation signaling using cAMP accumulation assays. The alanine mutation of D103^3.32^ eliminated amine-induced TAAR1 activation (Figure 2d,e and Table S3). Although the closest distance between Y294^7.43^ and METH was 4.4 Å, the alanine mutation of Y294^7.43^ eliminated the signaling. We found that D103^3.32^, Y294^7.43^, and the biogenic amine group formed a hydrogen network that was highly conserved in the aminergic receptors (Figure S2),^52−55^ indicating its important role in amine-induced aminergic receptor activation. S107^3.36^ also formed a hydrogen bond and hydrophobic interactions with D103^3.32^ and METH (Figure 2a). METH interacted with the surrounding residues via extensive aromatic (F186^ECL2^, W264^6.48^, F267^6.51^, and F268^6.52^) and hydrophobic (I104^3.33^, V184^ECL2^, I290^7.39^, and G293^7.42^) interactions. F186^ECL2^ and F267^6.51^ formed π–π interactions with the benzene ring (Figure 2a–c). Notably, the benzene ring was a necessary substituent of the TAAR1 agonists. The mutations of I104^3.33^, F186^ECL2^, W264^6.48^, F267^6.51^, and G293^7.42^ all failed to activate TAAR1 (Figure 2d,e and Table S3), whereas the alanine mutations of F268^6.52^ and V184^ECL2^ had little effect on the agonist potency (Figure 2d,e and Table S3).

### Structural Comparison of METH-TAAR1 Complex with PEA/AMPH/RO5236390-TAAR1 Complexes

PEA is an organic compound, classified as a natural monoamine alkaloid and trace amine that stimulants the human CNS. Reports suggest that PEA has therapeutic effects on mood and weight regulation.^56^ In the brain, PEA modulates monoaminergic neurotransmission by binding to TAAR1.^57^ A range of PEA derivatives, including AMPH, METH, and MDA, are formed by substituting one or more hydrogen atoms in the core structure of PEA.^58^ These derivatives serve as empathogens, stimulants, psychedelics, anorectics, bronchodilators, decongestants, antidepressants, etc. RO5256390 has a longer chemical backbone and higher activation efficiency than PEA, AMPH, and METH, making it a promising small-molecule treatment for METH addiction. To further explore the differences between these compounds, we conducted a more detailed comparative analysis of the structures of the METH-TAAR1 complex and the other three complexes.

**Figure 3:**
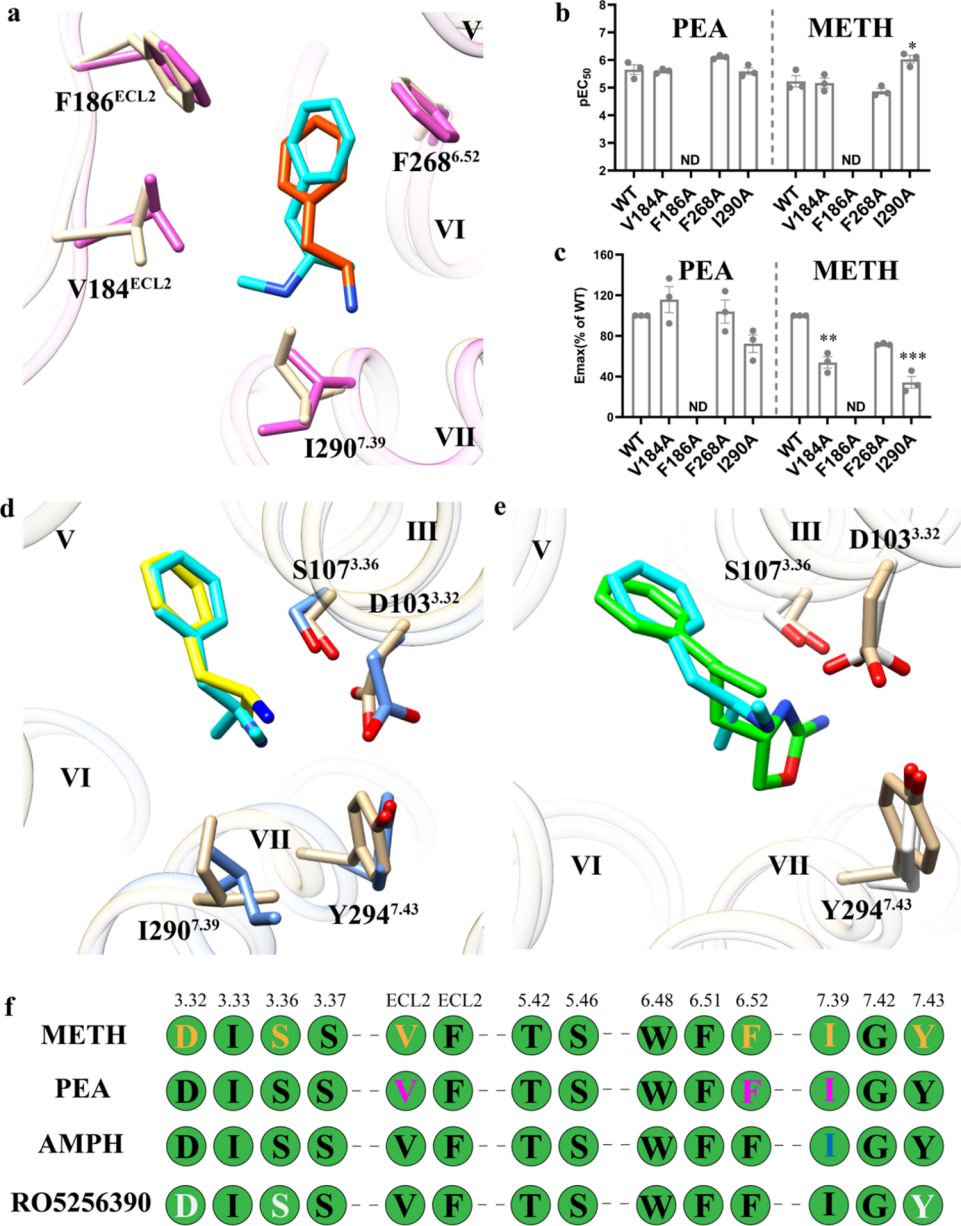
Comparison of METH and PEA/AMPH/RO5256390 recognition at TAAR1. (a, d, e) Comparison of the METH–TAAR1–G_s_ complex ((a) PDB ID: 9JKQ) with PEA–TAAR1–G_s_ complex, ((a) PDB ID: 8W89)/AMPH-TAAR1-G_s_ complex, ((d) PDB ID: 8JSO)/RO5256390-TAAR1-G_s_ complex, ((e) PDB ID: 8UHB). METH in METH–TAAR1–G_s_ complex colored cyan, TAAR1 in METH–TAAR1–G_s_ complex colored ivory. PEA in PEA-TAAR1-G_s_ complex colored orange-red; TAAR1 in PEA–TAAR1–G_s_ complex colored pink. AMPH in the AMPH-TAAR1-G_s_ complex is colored yellow; the TAAR1 in the AMPH-TAAR1-G_s_ complex is colored cornflower blue. RO5256390 in the RO5256390-TAAR1-G_s_ complex is colored green; TAAR1 in the RO5256390-TAAR1-G_s_ complex is colored light gray. (b, c) G_s_-cAMP accumulation results of WT TAAR1 and TAAR1 mutants activated by METH and PEA. Activities of ligands are identified as pEC_50_ (b) and *E*_max_ (c). Emax data are normalized to the percentage of the METH or PEA actives TAAR1. Data in (b, c) are mean ± s.e.m. of three independent experiments performed in technical triplicate. *P < 0.05, **P < 0.01, ***P < 0.001 (one-way ANOVA followed by Dunnett post test, compared with the response of the WT). ND, not detected. A detailed statistical evaluation is provided in Table S3. Source data are available as a Source Data file. (f) Comparison of different agonist binding sites between METH and PEA/AMPH/RO5256390. Residues that are special in interaction with METH are shown as cyan, and PEA/AMPH/RO5256390 are pink/blue/light gray. Residue positions (Ballesteros–Weinstein numbers) are indicated at the top of the scheme, respectively.

The structural comparison revealed that METH and PEA (PDB ID: 8W89) adopted a similar binding mode in the orthosteric site of TAAR1. METH had two more methyl groups than did PEA (Figure 3a). A comparative analysis of the amino acids in these regions indicated that different agonists induced changes in specific amino acid positions, resulting in variations in agonist potency. In the binding pocket, the modes of PEA and METH binding to TAAR1 were essentially identical, but there were three important differences. First, it is noteworthy that in both structures, ECL2 formed a loop covering the ligand-binding pocket (ECL2 loop), participating in ligand binding via hydrophobic interactions. Although F186^ECL2^ and V184^ECL2^ constitute the top of the ligand-binding pocket in METH-TAAR1, only F186^ECL2^ interacted with PEA in PEA-TAAR1. The F186^ECL2^A mutation eliminated TAAR1 activation by METH or PEA, and the V184^ECL2^A mutation reduced the METH-induced TAAR1 activation by half but did not affect PEA (Figure 3b,c and Table S3). This suggests the important roles of F186^ECL2^ and V184^ECL2^ in ligand binding and TAAR1 activation. Second, the F268^6.52^A mutation decreased the efficacy of METH but had little effect on PEA (Figure 3b,c and Table S3). This suggests that METH may have additional π–π interactions with F268^6.52^. Third, the I290^7.39^A mutation increased the half-maximal effective concentration (EC_50_) of METH by ∼6-fold but had a limited impact on the potency and efficacy of PEA. This suggests that the additional two methyl groups in METH compared to PEA occupied more space when binding to TAAR1, causing steric hindrance with I290^7.39^, thus affecting the binding and activation of TAAR1 by METH (Figure 3b,c and Table S3). These three important differences are likely one of the reasons for the distinct physiological functions observed between PEA and METH.

As expected, as a derivative of AMPH, METH exhibited a fundamentally similar binding mode (Figure 3d). The additional methyl group in METH likely created greater steric hindrance at position I290^7.39^, similar to what was observed with PEA. However, unlike METH, the additional 2-oxazoline group in RO5256390 inserted into the cavity formed by TM2, TM3, and TM7, creating a dense hydrogen bond network with D103^3.32^, S107^3.36^, and Y294^7.43^ (Figure 3e). This enhanced interaction likely contributed to the considerably higher activation potency of RO5256390 compared to PEA, AMPH, and METH.

### Structural Basis for the Stereoselective Binding Site of TAAR1 for AMPH-like Compounds

AMPH-like compounds (Figure S3), including METH, AMPH, MDA, and MDMA, are potent agonists of TAAR1.^18,20,59^ In addition, primate TAAR1 is a stereoselective binding site for AMPH-like compounds. The *S*(+) isomers of those AMPH-like compounds are reported to be more potent and efficacious than the *R*(−) isomers (Figure S3),^20,60^ indicating that they have similar molecular mechanisms for their binding and stereoselective characteristic.

We chose three representative molecules to investigate the stereoselective characteristics based on whether there was a substituent in the benzene ring head and the amino tail. To predict and compare the binding affinities of the *S* and *R* configurations, molecular docking and molecular dynamics simulations (MD) were performed for six systems. We first redocked the crystal ligand rigidly to the binding site to validate the accuracy of the molecule docking based on AutoDock Vina. The redocked conformations were aligned to the crystal structures, and the root-mean-square deviations (RMSDs) were compared. The predicted binding modes were the same as that of *S*-METH with a minor shift (Figure S4a), and the RMSD values were 0.74 Å (<2 Å), which showed that AutoDock Vina performed well. Based on the frequency and docking score of the ligand conformations, the initial pose of TAAR1 complexed with *S*-METH-, *R*-METH-, *S*-AMPH-, *R*-AMPH-, *S*-MDA- and *R*-MDA-bound structures. We compared the *S*-AMPH-TAAR1 complex structure (PDB ID: 8JSO) with our docking pose, the RMSD value was 0.80 Å (<2 Å) (Figure S4b), indicating the ligand binding modes were similar and the initial docking pose of *S*-AMPH was reliable. Then, we conducted a 120 ns MD simulation for each system (Figure S5). The initial docking poses were used as reference structures to observe the conformational changes of the molecules during the MD simulations. Initially, the *S* and *R* isomers were unstable, particularly at the benzyl position and the N-terminus (Figure S5). The benzyl carbons of *S*-AMPH and *S*-MDA shifted from pointing to TM6 to pointing to the extracellular side at the benzyl position. It was subsequently redirected to helix 6 and continued for 120 ns. The benzyl position of *S*-METH underwent conformational changes from pointing to TM6 to pointing to the extracellular side and then to TM3 stably. The N-terminus of the *S* isomers extended further toward TM3 in one frame during the MD simulation, with a corresponding shift of residue S107^3.36^ (Figure S5a,e,i) and often occurred in the remaining simulations. The benzyl position of *R*-AMPH and *R*-MDA shifted from pointing to TM6 to pointing to the extracellular, and pointed to TM3 steadily, whereas *R*-METH always faced TM6. The N-terminus of the *R* isomers was away from TM3 in one frame, which almost continued to the final frame (Figure S5c,g,k). Overall, the N-termini of the *S* and *R* isomers had different interactions with TM3, which may play an important role in stereoselectivity.

**Figure 4:**
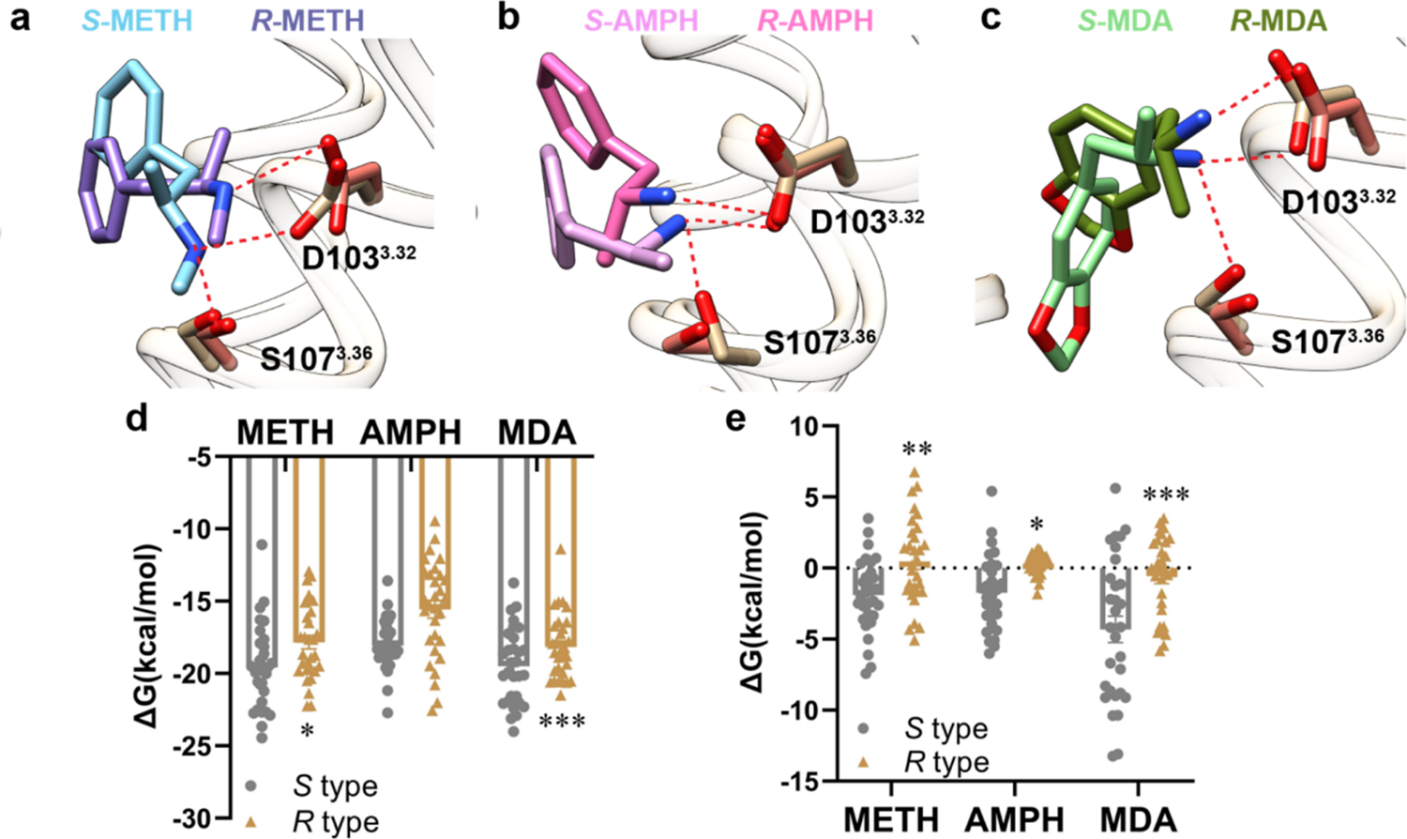
Comparison of *S* and *R* configurations bound to TAAR1. (a–c) Conformations alignment of *S*-METH and *R*-METH (a), *S*-AMPH and *R*-AMPH (b), and *S*-MDA and *R*-MDA (c). The complex structures were from the last frame. (d) Comparison of total binding free energy of *S* configuration and *R* configuration using the MM/PBSA method. The binding free energy of the *S*-isomer is better than the *R*-isomer. (*n* = 30, unpaired, two-way ANOVA followed by Bonferroni test, compared with *S* type, *P < 0.05, **P < 0.01, ***P < 0.001). Data are presented as mean ± s.e.m. (e) Energy decomposition of residue S107^3.36^. S107^3.36^ contributes more energy in *S*-isomer bound structures than in *R*-isomer bound structures. *n* = 30, unpaired, two-way ANOVA followed by Bonferroni test, compared with *S* type, *P < 0.05, **P < 0.01, ***P < 0.001. Data are presented as mean ± s.e.m. Brown, main chain. The residues are colored as brown and salmon of *S* and *R* configuration bound TAAR1, respectively; Cyan, *S*-METH; purple, *R*-METH; pink, *S*-AMPH; hot pink, *R*-AMPH; lime, *S*-MDA; olive, *R*-MDA. The salt-bridge interactions and hydrogen bond interactions are all shown as the red dashed line. A detailed statistical evaluation is provided in Table S4. Source data are available as a Source Data file.

To verify our hypothesis, the molecular mechanics-Poisson–Boltzmann surface area (MM-PBSA) was calculated. A stable segment of the MD trajectory encompassing 90–120 ns was selected as a 30 ns frame for the MM-PBSA estimation. The binding free energy of the *S*-isomer was better than that of the *R*-isomer (Figure 4d and Table S4), which was consistent with the experiments. Subsequently, to determine which residues contributed to the configuration selectivity, we combined the binding poses and interaction analyses (Figures 4a–c, S5, and S6). The *S* and *R* isomers each formed a stable salt-bridge interaction with residue D103^3.32^. Although the *R* configurations formed hydrogen bonds with S107^3.36^ in certain frames, the frequency of interactions was less than the *S* isomers (Figures S5c,g,k and S6). The alanine mutation of S107^3.36^ eliminated METH-induced TAAR1 activation (Figure 2d,e and Table S3). Moreover, further energy decomposition results showed that S107^3.36^ in the *S*-isomer contributed more to the molecule binding affinity (Figure 4e and Table S4), indicating the important role of S107^3.36^ in the configuration selectivity for the isomers bound to TAAR1.

### Structural Basis for TAAR1 Activation Mechanism by METH

**Figure 5:**
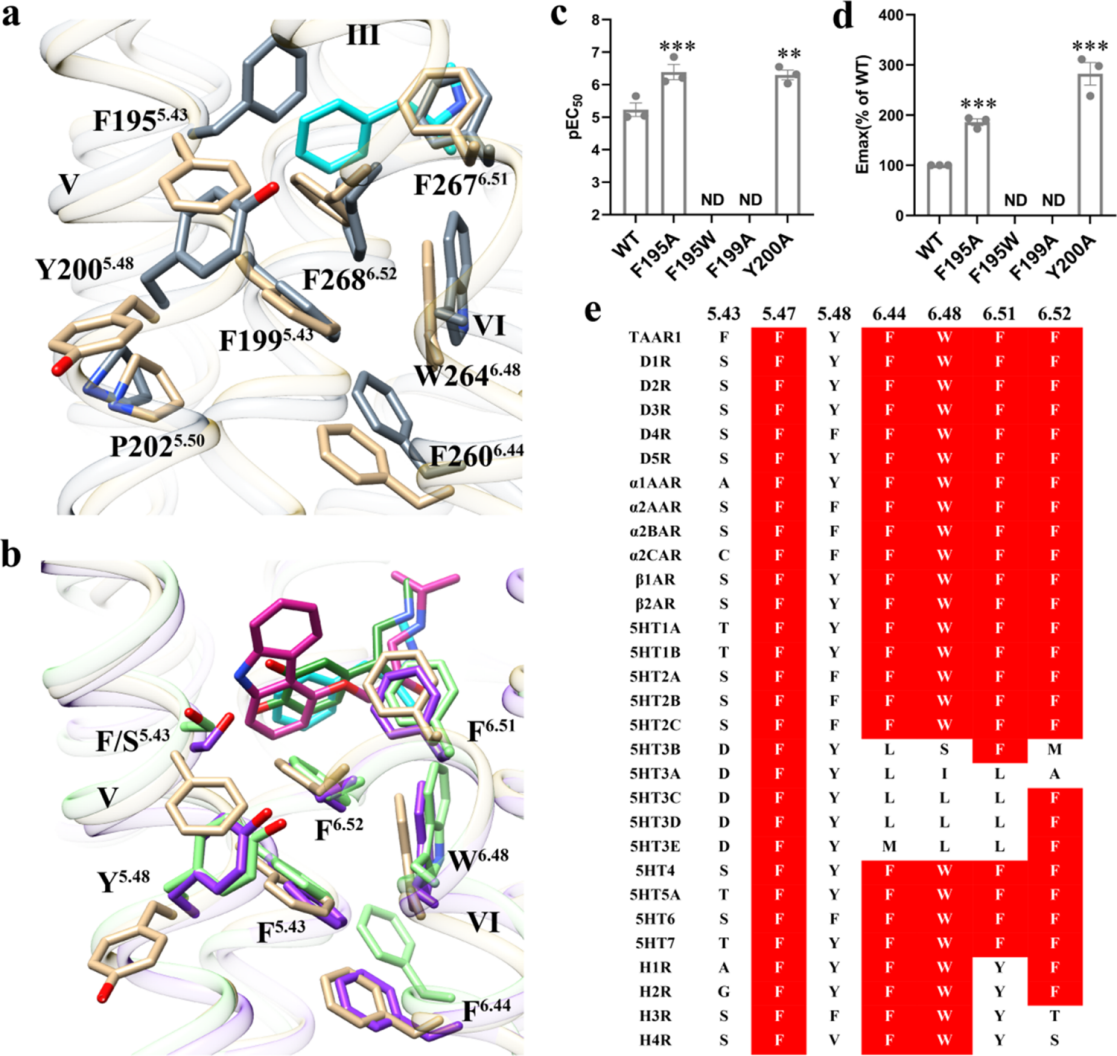
Hydrophobic core in TAAR1. (a) Comparison of active TAAR1 (ivory, PDB ID: 9JKQ) with AlphaFold 2.0-predicted TAAR1 (gray). (b) Comparison of active TAAR1 (ivory) with inactive β2AR (PDB ID: 2RH1, light green) and active β2AR (PDB ID: 4LDO, purple). (c, d) G_s_-cAMP accumulation results of WT TAAR1 and TAAR1 mutants activated by METH. Activities of METH are identified as pEC_50_ (c) and *E*_max_ (d). Emax data are normalized to the percentage of the reference agonist METH. Data in (c, d) are mean ± s.e.m of three independent experiments performed in technical triplicate. *P < 0.05, **P < 0.01, ***P < 0.001 (one-way ANOVA followed by Dunnett post test, compared with the response of the WT). ND, not detected. A detailed statistical evaluation is provided in Table S3. Source data are available as a Source Data file. (e) Comparison of hydrophobic core between TAAR1 and anther amine receptors. Conserved residues are labeled with red, respectively. Residue positions (Ballesteros–Weinstein numbers) are indicated at the top of the scheme, respectively.

The common phenyl groups of agonists were linked to the hydrophobic core formed by F195^5.43^, F199^5.47^, Y200^5.48^, F267^6.51^, F268^6.52^, W264^6.48^, and F260^6.44^ via hydrophobic interactions. All of the residues were highly conserved in all amine receptors except F195^5.43^, most of which were hydrophilic acids such as S^5.43^/T^5.43^ (Figure 5e). We compared the experimentally determined active TAAR1 with the AlphaFold 2.0-predicted TAAR1 under ligand-free conditions without any interacting proteins. Across the comparisons, repacking occurred in all of the hydrophobic core residues. The hydrophobic network caused W^6.48^ to pack against F^6.44^, resulting in the reorganization of the transmembrane segments upon agonist binding. Interestingly, F195^5.43^ exhibited the most substantial conformational changes from the predicted apo state to the active state (Figure 5a), indicating that this residue is important in receptor activation. In the predicted apo state, F195^5.43^ pointed toward the center of the helical bundle and the orthosteric binding pocket (hereafter referred to as F195^5.43^-in), while in the active state, the agonist binding pushes F195^5.43^ out to face the lipid (F195^5.43^-out) (Figure 5a). In addition, mutation F195^5.43^W, with a larger side chain, inhibited agonist-induced receptor activation (Figure 5c,d). The larger side chain may have occupied the binding pocket space and hindered agonist-induced TM5 rearrangement. Mutation F195^5.43^A revealed a substantial increase in the level of METH-induced TAAR1 activation. This may have occurred because the smaller side chain made it easier to change its conformation from F195^5.43^-in to F195^5.43^-out (Figure 5c and Table S3). Although repacking of the hydrophobic core was also observed between the inactive and active β2AR structures, their activation mechanisms differed. Additionally, the hydrogen bonds among adrenaline and S^5.42^ and S^5.46^ in β2AR resulted in the inward movement of P^5.50^, rearrangement of the P^5.50^I^3.40^F^6.44^ motif, and opening of the intracellular side of TM6, which is a common activation mechanism for aminergic receptors. However, the inward movement of TM5 in the ligand-binding pocket did not occur in the agonist-bound TAAR1 structures compared to inactive β2AR or the AlphaFold 2.0-predicted TAAR1 (Figure 5b). This illustrates that TAAR1 adopted different activation mechanisms than aminergic receptors.^49,52,61,62^ A series of conformational changes in F195^5.43^, F199^5.47^ and Y200^5.48^ caused the inward movement of P^5.50^. In addition, agonist binding and residue rearrangement in the hydrophobic core of TM5 lead to conformational changes in the TM6 residues F267^6.51^, F268^6.52^, W264^6.48^, and F260^6.44^, allowing the intracellular side of TM6 to open and engage the G_s_ protein.

The structure of the TAAR1-G_s_ complex provides an important model for analyzing the interactions between GPCRs and G proteins. The interface between TAAR1 and G_s_ includes ICL2 of TAAR1, the cytoplasmic sides of TM3, TM5, TM6, and helix 8, as well as the Gα_s_ α5 helix, an αg-α4 loop, and αN. Upon TAAR1 activation, the α5 helix of Gα_s_ penetrates the cavity formed by TM3, TM5, and TM6. The C-terminal end of helix α5 in Gα_s_ forms extensive contacts with the hydrophobic surface created by the cytoplasmic ends of TM3, TM5, TM6, and the initial helix 8 segment. This binding induces a conformational change in the G_s_. Although ICL1 does not directly interact with the G proteins, ICL2 is buried in a hydrophobic pocket formed by Gα_s_ αN, β1, and α5, establishing strong hydrophobic interactions. This binding mode is relatively conserved among the Class A GPCRs.

## Discussion

Although the mechanisms underlying METH-induced addiction are not yet fully understood, previous studies have reported that METH activates TAAR1, causing a series of downstream signaling changes. METH activates TAAR1 by binding to it on the cell membrane or by entering the cell through the dopamine transporter (DAT) and binding to intracellular TAAR1, which leads to various responses, such as upregulating the trace amines levels in the cytoplasm.^51^ Subsequently, these elevated amine concentrations further activate intracellular TAAR1, triggering downstream protein kinase A (PKA)/protein kinase C (PKC) signaling pathways.^63^ Phosphorylation of DAT induced by these pathways leads to the internalization of DAT, which reduces the cellular monoamine content. Moreover, PKC-mediated phosphorylation induces the reverse transport function of DAT, resulting in DA efflux.^20^ Because DAT is found at neuronal synapses as well as in other cellular regions, effluxed DA may disperse to other areas, decreasing the DA concentration at synaptic sites and subsequently reducing neuronal firing rates.^29^ In neurons that do not express TAAR1, METH competes with DA for binding to DAT, entering the cell and inhibiting DAT reuptake of DA.^51,64^ Moreover, METH leads to a substantial amount of DA released into the synaptic cleft by exocytosis, inducing a state of cellular hyperexcitability and increasing neuronal firing rates.^63,65^ When the DA concentration in the synaptic cleft becomes excessively high, activated TAAR1 prevents the cells from entering an abnormal excited state.

In addition to DA, other neurotransmitters are affected by METH use, including serotonin and the norepinephrine (NE) system. METH is currently believed to act primarily via mechanisms similar to those affecting the dopaminergic system. These mechanisms include inhibiting norepinephrine transporter (NET) and serotonin transporter (SERT) from reuptaking monoamines, inducing their reverse transport, and promoting the release of vesicular monoamines,^66−68^ thereby increasing the monomine concentration in the synaptic cleft. However, the precise mechanisms by which METH regulates serotonin and NE systems remain unclear. Interestingly, the affinity of METH for NET is 5–9 times higher than for DAT,^69^ and NE greatly increases the release of DA.^70^ Compared to the acute response of the dopaminergic system to METH, the serotonergic system undergoes partial but persistent functional loss in several brain regions, such as the striatum, cortex, and hippocampus,^71^ when exposed to high METH concentrations over a long period. This may be one of the reasons for METH-induced neurotoxicity in the monoaminergic system.

To further explore the molecular mechanism by which METH activates TAAR1, we analyzed cryo-EM structures of the METH–TAAR1–G_s_ signaling complex, highlighting distinct binding modes among different ligands. By investigating the binding pocket of TAAR1, we found that D103^3.32^, S107^3.36^, and Y294^7.43^ form a conserved hydrogen bond network with METH, whereas F186^ECL2^, W264^6.48^, F267^6.51^, and F268^6.52^ engage in π–π stacking interactions with METH. Additionally, I104^3.33^, V184^5.52^, I290^7.39^, and G293^7.42^ form hydrophobic interactions with METH. These interactions stabilize the binding of ligands within the pocket (Figure 2). We also observed that METH interacts more extensively with V184^ECL2^, F268^6.52^, and I290^7.39^ in the binding pocket of TAAR1 compared to PEA. This is attributed to the two additional methyl groups present in METH. The potency of METH is 2.6 times lower than that of PEA (Table S3), indicating that METH likely generates greater steric hindrance within the binding pocket, resulting in less tight binding compared to PEA. Our structure reveals the common ligand-binding modes in the orthosteric binding pocket of TAAR1, as well as the key residues involved in ligand selectivity.

Recent studies have shown that AMPH-like psychoactive drugs have therapeutic potential, showing considerable promise in treating mental disorders. It is currently believed that AMPH-like compounds directly influence DA signaling via TAAR1,^72^ contributing to the onset of psychotic symptoms. Considering the presence of chiral isomers in AMPH-like compounds, we conducted MD simulations on AMPH, METH, and MDA. The results are consistent with findings reported in the literature: *S*-type compounds exhibit lower energy and form more stable bindings with TAAR1 than *R*-type compounds. Furthermore, our results elucidate the important role of S107^3.36^ in the selection of different isomers of AMPH-like compounds. Nevertheless, the mechanism of action of AMPH-like compounds still requires further investigation.

Earlier literature mainly described the activation mechanism of TAAR1.^73,74^ However, with in-depth research on TAAR1, we discovered a more specific activation mechanism of TAAR1. Compared to β2AR, TAAR1 forms a tighter hydrophobic core on TM5 and TM6, including residues F195^5.43^, F199^5.47^, Y200^5.48^, F267^6.51^, F268^6.52^, W264^6.48^, and F260^6.44^. In the inactive state, F195^5.43^ is oriented toward the interior of the helix; however, following receptor activation, F195^5.43^ flips outward. Mutating F195^5.43^ to alanine, which has a smaller side chain, increases METH/PEA activation by nearly 2-fold, whereas mutating F195^5.43^ to tryptophan, which has a larger side chain, inhibits METH/PEA activation. Similarly, increased activation was observed in the Y200^5.48^A mutant, suggesting the crucial importance of this hydrophobic “core” for the specificity and functionality of TAAR1. These data contribute to a better understanding of the activation mechanism of TAAR1, offering additional insights into the activation mechanism of TAAR1.

In summary, our work reveals the molecular basis of METH recognition by TAAR1 and the possible mechanisms of binding of chiral molecules of AMPH-like psychoactive drugs to TAAR1. Additionally, we discovered a hydrophobic core comprising residues with benzene rings on TM5 and TM6, further explaining the unique mechanism of TAAR1 activation. This provides novel insights into the interaction between TAAR1 and monoaminergic system signaling, as well as for the design of the dopaminergic system. This research provides valuable guidance for the future development of psychotropic drugs.

## Methods

### Construct

The human TAAR1 was modified to contain a hemagglutinin (HA) signal peptide and a thermally stabilized bRIL at the N-terminus, a flag-tag, and a strep-tag at the C-terminus. Two mutations (H63^2.44^V, F112^3.41^W) were to improve protein yield. A dominant negative Gα_s_ (DNGα_s_) construct was generated by site-directed mutagenesis to incorporate eight mutations including S54N, G226A, E268A, N271 K, K274D, R280 K, T284D, and I285T.^75^

### Insect Cell Expression

Human TAAR1, DNGα_s_, and His6-tagged human Gβ_1_ and Gγ_2_ were coexpressed in HighFive insect cells (Invitrogen) using the Bac-to-Bac Baculovirus Expression System (Invitrogen). Cell cultures were grown to a density of 1.5–2 million cells per milliliter and then infected with high-titer viral stocks at a multiplicity of infection (MOI) ratio of 0.5:1:1 for TAAR1, DNGα_s_, and Gβ_1_γ_2_. Cells were collected by centrifugation 48 h after infection and stored at −80 °C until use.^76^

### Purification of METH-TAAR1-G_s_ Complexes

Cells were suspended in a buffer including 20 mM HEPES, pH 7.4, 50 mM NaCl, and 2 mM MgCl_2_ supplemented with protease inhibitor cocktail tablets (Roche). TAAR1-G_s_ complex was obtained by adding 10 μM METH (Sigma), 10 μg mL^–1^ Nb35 (prepared as previously described^77^), and 25 mU mL^–1^ Apyrase; followed by 1 h incubation at 20 °C. Insoluble material was removed by centrifugation at 30,000*g* for 30 min. The complex protein was solubilized in 25 mM HEPES, pH 7.4, 150 mM NaCl, 0.5% (w/v) lauryl maltose neopentyl glycol (LMNG, Anatrace), 0.025% cholesterol hemisuccinate (CHS, Anatrace), 2 mM MgCl_2_, 25 mU mL^–1^ Apyrase, and 10 μM METH at 4 °C for 2 h. The supernatant was isolated by centrifugation and was further incubated with Strep-Tactin XT (IBA) resin overnight at 4 °C.

The resin was washed with 20 column volumes of 25 mM HEPES, pH 7.4, 150 mM NaCl, 0.01% (w/v) LMNG, 0.0005% CHS, 2 mM MgCl_2_, and 10 μM METH. Then, the resin was eluted with five column volumes of 150 mM Tris-HCl, pH 8.0, 150 mM NaCl, 0.01% (w/v) LMNG, 0.0005% CHS (Anatrace), 2 mM MgCl_2_, 50 mM biotin, and 10 μM METH. The complex protein was then purified by size-exclusion chromatography using a Superdex 200 Increase 10/300 column (GE Healthcare) preequilibrated with 20 mM HEPES, pH 7.4, 100 mM NaCl, 0.01% (w/v) LMNG, 0.0005% CHS, 2 mM MgCl_2_, and 5 μM METH.

### Cryo-EM Grid Preparation and Data Collection

For grid preparation, a sample (3 μL) of purified TAAR1-METH complexes was loaded onto glow-discharged 300-mesh Au holey carbon grids (R1.2/1.3, Quantifoil) at 4 °C at 100% humidity. Grids were plunge-frozen into liquid ethane with 5 s wait time, 0 blot force, and a blot time of 3 s using Vitrobot Mark IV (Thermo Fisher Scientific).

Data sets of TAAR1-METH complex were collected using a 300 keV Titan Krios electronic microscope equipped with Gatan K3 direct electron detector and GIF Quantum energy filter. Movies were recorded using EPU in super-resolution mode with a binning of 2 and defocus range from −1.0 to −1.5 μm at a nominal magnification of 105,000×, resulting in a calibrated pixel size of 0.851. The total dose was 54 electrons per Å2 fractions, which was fractioned to 40 frames.

### Data Processing

3824 movies were collected for TAAR1-METH and aligned using MotionCor in Relion and exported to CryoSPARC for Contrast Transfer Function and following processing. 4,284,829 particles were template-picked, and the best class were selected after two rounds of ab initio refinement and heterogeneous refinement. Final data sets of 647,397 particles were reextracted and subjected to nonuniform refinement, corresponding to a final resolution of 2.66 Å.

### Model Building and Refinement

The initial TAAR1 model was created by AlphaFold 2.0, and the GPR119-G_s_ complex (PDB ID: 7WCM) was used as the starting model of the G protein complex. The models were rigid-fitted into EM density map of TAAR1-METH complex using UCSF Chimera and adjusted with iterations of manual refinement in Coot and refinement in Phenix. The ligand model of METH was generated with elBow in Phenix, docked into EM density maps in Coot, and refined in Phenix. Structural figures were displayed by UCSF Chimera.

### Flow Cytometry

The cell surface TAAR1 expression level was detected by incubating 10 μL of cells with 10 μL of monoclonal anti-FLAG M2–fluorescein isothiocyanate antibody (Sigma-Aldrich) at 4 °C for 20 min in the dark. The fluorescent signal of the bound antibody was measured using a FACSCalibur instrument (Becton Dickinson, Sunnyvale, CA). Single-parameter histograms can be used to further identify distinct cell types that have an antibody-specific population of cells. Cells expressed in TAAR1 were gated according to negative cells without fluorescein isothiocyanate.

### cAMP Assay

HEK293 cells (Invitrogen) were harvested 48 h after transfection with 1 μg of mL^–1^ plasmid. cAMP accumulation was measured using an HTRF cAMP kit (Cisbio Bioassays, 62AM6PEC) according to the manufacturer’s instructions. In brief, the HEK293 cells expressing TAAR1 were seeded onto 384-well plates (5 μL, 8000 cells per well) and incubated at 37 °C for 30 min with different concentrations of METH (10^–4^–10^–10^ M). Then, 5 μL of detection reagent d2-conjugated cAMP and 5 μL of cryptate (Eu)-conjugated antibody were added in each well. After incubation at room temperature for 1 h, the plates were read using a microplate reader (PerkinElmer) with excitation at 330 nm and emission at 620 and 665 nm. cAMP accumulation was analyzed by a standard dose–response curve using GraphPad Prism 9.0 (GraphPad Software). Emax ± s.e.m. and pEC_50_ ± s.e.m. were calculated by using nonlinear regression (curve fit).

### Protein–Ligand Docking

The crystal structure of methylamphetamine combined with TAAR1 was first preprocessed using PyMOL (https://pymol.org/2/), and the nanobody was removed. Docking input files were generated using AutoDockTool (version 1.5.6).^78^ The protein was added to hydrogen atoms and computed Gasteiger charges. Then, it was saved in PDBQT format. The binding site was centered on methylamphetamine, the central coordinate was (*X* = 134.75, *Y* = 135.70, *Z* = 102.99) for TAAR1 and the grid dimensions were 60 × 60 × 60 Å^3^. The amine agonists, *S*-METH/*R*-METH/*S*-AMPH/*R*-AMPH/*S*-MDA/*R*-MDA, were added hydrogen, calculated Gasteiger charges, assigned rotatable bonds, and converted into PDBQT format. AutoDock Vina (version 1.1.2) was selected to conduct molecule docking.^79^ In the docking process, the parameter exhaustiveness was set to 24, and 20 conformations were generated for each ligand. The most reliable binding poses were selected as the initial structures for further MD simulation analysis according to the visual inspections and favorable interaction energy.

### Molecular Dynamics Simulations

MD simulations were performed using the GROMACS 2022.6 package.^80^ The complex was embedded in a preequilibrated palmitoyl oleoylphosphatidyl choline (POPC) bilayer using the CHARMM-GUI.^81^ CHARMM36m force field^82^ was used for the protein, POPC, ions, and water molecules. Before the MD simulations, the protonation states of the His residues were first determined by the H++ website.^83^ The topology parameters of ligands were generated through CHARMM Generalized Force Field (CGenFF) program.^84^ The 6 systems were then solvated in the same box size (100 × 100 × 114 Å^3^) with TIP3P waters and added counterions (Na^+^ and Cl^–^) in order to neutralize the charges and simulate a physiological environment of 0.15 M NaCl. The solvated systems were then subjected to energy minimization using the steepest descent algorithm. The systems were equilibrated with the v-rescale^85^ thermostat at 310 K and semi-isotropic Berendsen^86^ barostat at 1 bar in the NPT ensemble. For the production phase, semi-isotropic Parrinello–Rahman coupling^87^ at 1 bar was used. Each system performed 100 ns production runs in the NPT ensemble with a time step of 2 fs, the LINCS algorithm^88^ was used to constrain bond lengths. The particle mesh Ewald (PME)^89^ method was used to treat the long-range electrostatic interactions. The cutoff for the short-range interactions was set to 1.2 nm. The results were analyzed using PyMOL, GROMACS tools, and in-house scripts. The binding free energy of each protein–ligand complex was calculated with gmx_MMPBSA(version 1.6.2)^90^ based on MM-PBSA.py^91^ in AmeberTools23.

## Supporting Information Available

Engineered TAAR1 construct cAMP signaling assays; alignment of the cryo-EM structures and docking poses; cryo-EM data collection, model refinement, and validation statistics; TAAR1 mutants construct cAMP signaling assays; description of cAMP assay; comparison of amine receptor structures; different amphetamine-like drugs; molecular dynamics simulations results; and structure data processing model building and refinement (PDF)
Models (ZIP)
Source data of cAMP assay and binding free energy (XLSX)

## Abbreviations Used

ADHD: attention-deficit hyperactivity disorder
AMPH: amphetamine
BRIL: apocytochrome b562 fusion protein
cAMP: cyclic adenosine monophosphate
CNS: central nervous system
cryo-EM: cryo-electron microscopy
D1R: dopamine receptor
DA: dopamine
DAT: dopamine transporter
EC_50_: half-maximal effective concentration
ECL: extracellular loop
GPCR: G protein-coupled receptor
ICL: intracellular loop
MD: molecule docking
MDA: 3,4-methylenedioxyamphetamine
MDMA: 3,4-methylenedioxymethamphetamine
METH: methamphetamine
MOI: multiplicity of infection
NE: norepinephrine
NET: norepinephrine transporter
NB35: nanobody 35
ND: not detected
OA: octopamine
PEA: β-phenylethylamine
PKA: protein kinase A
PKC: protein kinase C
PTSD: post-traumatic stress disorder
RESD: root-mean-square deviation
SET: serotonin transporter
TAAR1: trace amine-associated receptor 1
TM: transmembrane helices
TYR: tyramine
WT: wild type
β2AR: β-2 adrenergic receptor
