# Supplementary material for: Molecular Mechanisms of Methamphetamine-Induced Addiction via TAAR1 Activation
Source: J Med Chem. 2024 Oct 2;67(20):18593–605. doi: 10.1021/acs.jmedchem.4c01961 (PMC11513891; doi:10.1021/acs.jmedchem.4c01961)
Supplement: Supplementary file 1 — jm4c01961_si_001.pdf [file jm4c01961_si_001.pdf]

Molecular Mechanisms of Methamphetamine-Induced Addiction via TAAR1 Activation

Yun Lin[1#], Jiening Wang[2#], Fan Shi[3#], Linlin Yang[3*], Shan Wu[2*], Anna Qiao[1*], Sheng Ye[1*]

1 Tianjin Key Laboratory of Function and Application of Biological Macromolecular Structures, School of Life Sciences, Tianjin University, 92 Weijin Road, Nankai District, Tianjin 300072, China;

2 State Key Laboratory of Biocatalysis and Enzyme Engineering, Hubei Collaborative Innovation Center for Green Transformation of Bio-Resources, Hubei Key Laboratory of Industrial Biotechnology, School of Life Sciences, Hubei University, Wuhan, Hubei 430062, China;

3 Department of Pharmacology, School of Basic Medical Sciences, Zhengzhou University, Zhengzhou, Zhengzhou 450001, China.

Corresponding Authors Information:

Sheng Ye, School of Life Sciences, Tianjin University, 92 Weijin Road, Nankai District, Tianjin 300072, China; E-Mail: sye@tju.edu.cn

Anna Qiao, School of Life Sciences, Tianjin University, 92 Weijin Road, Nankai District, Tianjin 300072, China; E-mail: anna.qiao@tju.edu.cn

Shan Wu, School of Life Sciences, Hubei University, 368 Youyi Avenue, Wuchang District, Wuhan, Hubei 430062, China; E-mail: wushan91@hubu.edu.cn

Linlin Yang, School of Basic Medical Sciences, Zhengzhou University, 100 Science Road Zhongyuan District, Zhengzhou, Henan 450001, China; E-mail: yanglin-89@zzu.edu.cn

Authors Information:

Yun Lin–Tianjin Key Laboratory of Function and Application of Biological Macromolecular Structures, School of Life Sciences, Tianjin University, 92 Weijin Road, Nankai District, Tianjin 300072, China;

Jiening Wang–State Key Laboratory of Biocatalysis and Enzyme Engineering, Hubei Collaborative Innovation Center for Green Transformation of Bio-Resources, Hubei Key Laboratory of Industrial Biotechnology, School of Life Sciences, Hubei University, Wuhan, Hubei 430062, China;

Fan Shi–Department of Pharmacology, School of Basic Medical Sciences, Zhengzhou University, Zhengzhou, Zhengzhou 450001, China.

# These authors contribute equally to this work.

Figure S1 | Engineered TAAR1 construct cAMP signaling assays. S3

Figure S2 | Conserved Hydrogen Bond Network in Amine Receptors. S4

Figure S3 | Chiral Structure and EC_{50} of Classic Amphetamine-like Drugs. S5

Figure S4 | Alignment of the cryo-EM structures and docking poses. S6

Figure S5 | Ligand RMSD and conformational changes along the MD simulations. S7

Figure S6 | Ligand hydrogen bonds interactions. S9

Figure S7 | Structure determination of METH bound TAAR1–G_s complexes. S10

Figure S8 | Cryo-EM density maps and models of TM1-7, and Helix8 of TAAR1. .... S11

Table S1 | Cryo-EM data collection, model refinement and validation statistics S12

Table S2 | Engineered TAAR1 construct cAMP signaling assays S13

Table S3 | TAAR1 Mutants construct cAMP signaling assays S14

Table S4 | The binding free energies of the *S*- and *R*-configurations of AMPH/ METH/ MDA ... S16

Figure S1 | Engineered TAAR1 construct cAMP signaling assays.

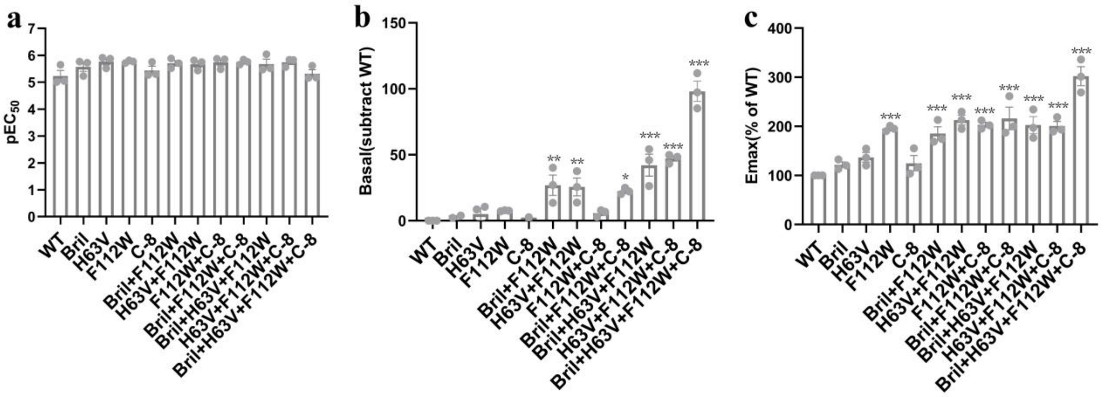

a, **b**, **c**, G_s–cAMP accumulation results of WT TAAR1 and TAAR1 mutants activated by METH. Activities of ligands are identified as pEC_{50} (**a**) , Basal (**b**) and Emax (**c**). Basal data are subtract WT, and Emax data are normalized to the percentage of the METH actives TAAR1. Data in **a**, **b**, **c**, are mean±s.e.m of three independent experiments performed in technical triplicate. *P < 0.05, **P < 0.01, ***P < 0.001, (one-way ANOVA followed by Dunnett post-test,compared with the response of the WT). ND, not detected. A detailed statistical evaluation is provided in Table S2. Source data are available as a Source Data file.

Figure S2 | Conserved Hydrogen Bond Network in Amine Receptors.

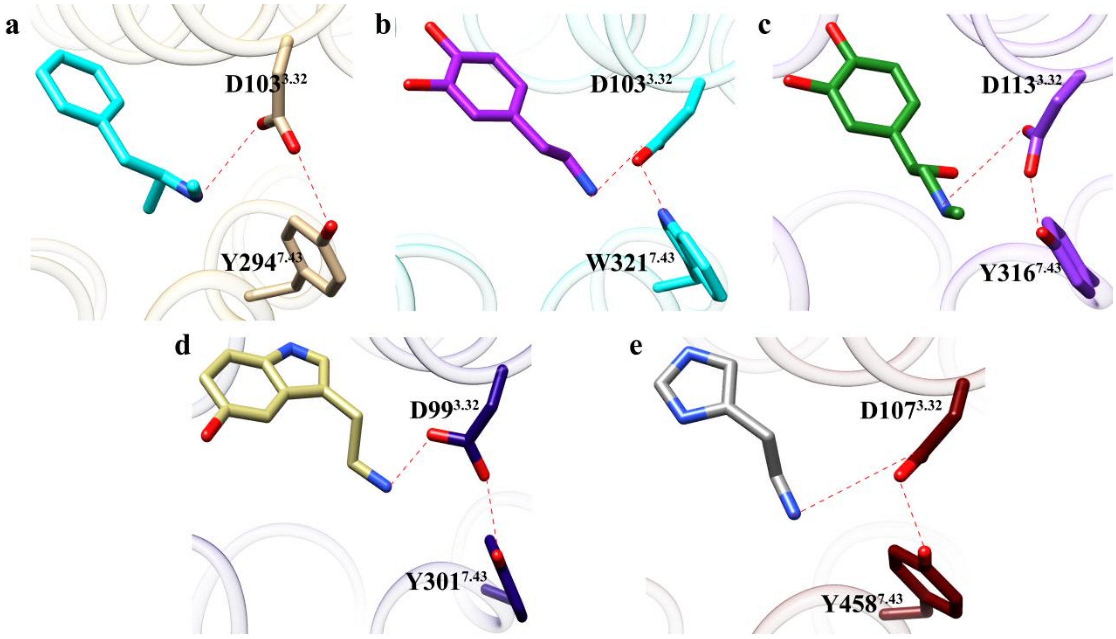

a, METH (cyan) and TAAR1 (ivory, PDB ID: 9JKQ). **b**, DA (purple) and D1R (cyan, PDB ID: 7CKZ). **c**, Adrenaline (forest green) and β2AR (dark magenta PDB ID: 4LDO), **d**, Serotonin (dark khaki) and 5HT4R(dark purple PDB ID: 7XT8). **e**, Histamine (gray) and H1R (brown PDB ID: 7DFL).

Figure S3 | Chiral Structure and EC_{50} of Classic Amphetamine-like Drugs.

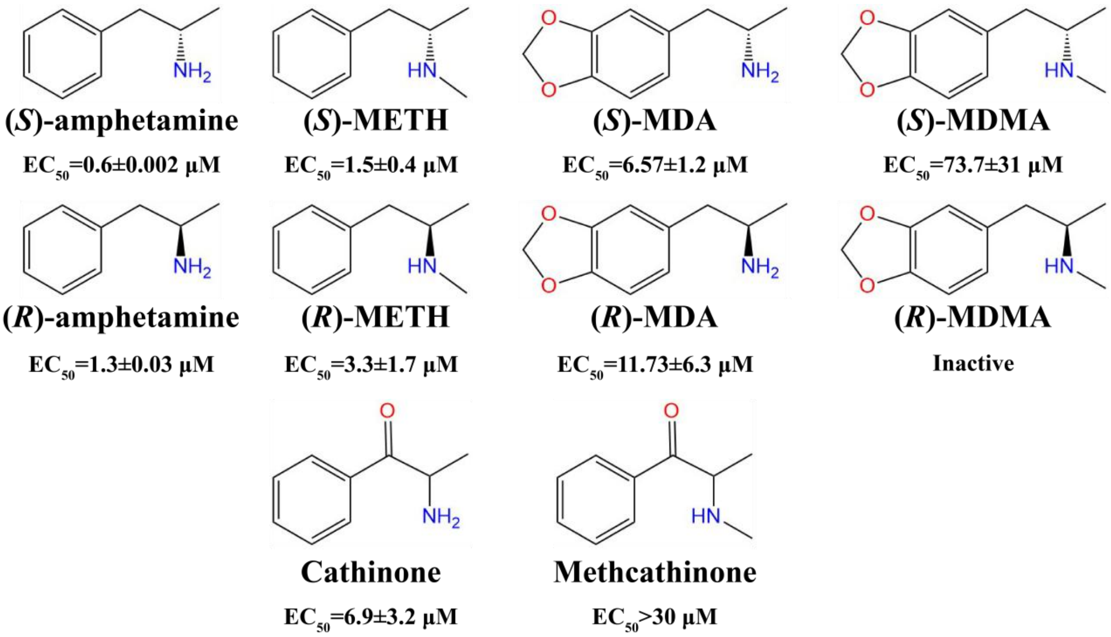

METH: methamphetamine; MDA: 3,4-methylenedioxy- amphetamine; MDMA: 3,4-methylenedioxymethamphetamin

Figure S4 | Alignment of the cryo-EM structures and docking poses.

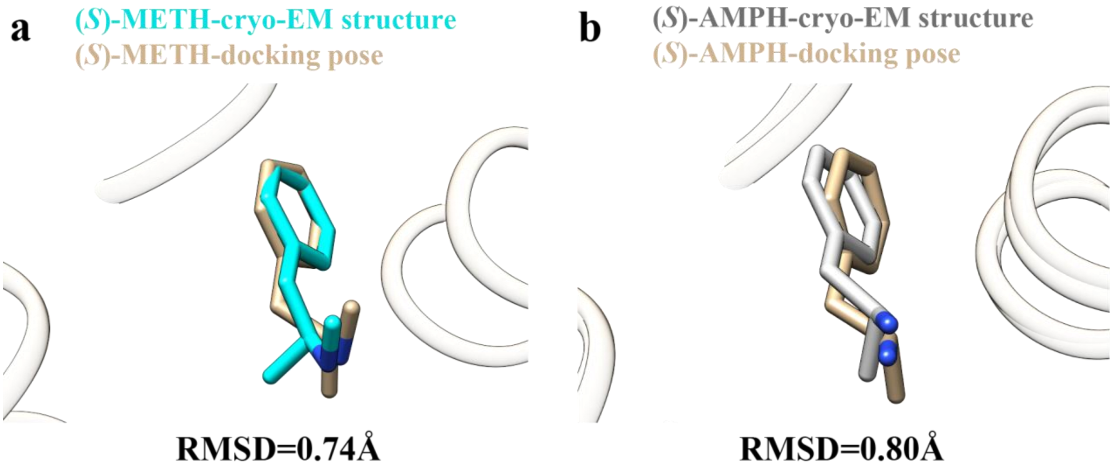

a, b *S*-METH and *S*-AMPH were redocked into the orthosteric pocket and compared the docking pose with the cryo-EM structure respectively. Brown, protein TAAR1 **(a,** PDB ID: 9JKQ**);** Brown, docking poses **(b)**; Cyan, cryo-EM structure of *S*-METH **(a)**; Gray, cryo-EM structure of *S*-AMPH **(b,** PDB ID: 8JSO**).**

Figure S5 | Ligand RMSD and conformational changes along the MD simulations.

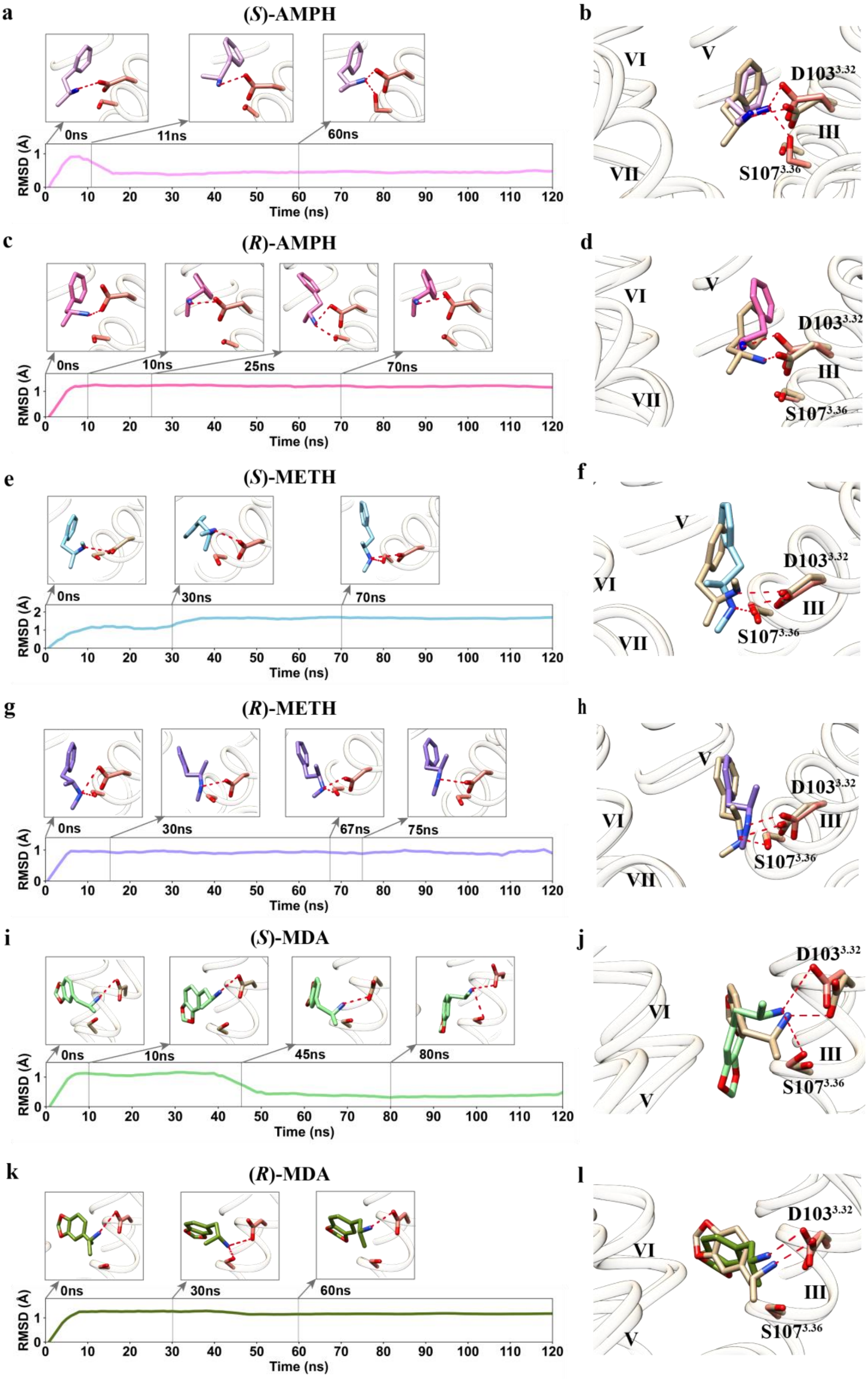

a, c, e, g, i, k, 120ns MD simulations were conducted and the ligand root mean square deviation (RMSD) levels were calculated and using docking results as the reference. The conformational changes were shown at different frames. **b, d, f, g, h, j**. Brown, protein TAAR1; Brown, docking poses; Blue, *S*-METH; Purple, *R*-METH; Pink, *S*-AMPH; Hot pink, *R*-AMPH; Lime, *S*-MDA; Olive, *R*-MDA. The ligand RMSD colors correspond to their ligand colors. The salt-bridge interactions and hydrogen bond interactions are all shown as red dash lines.

Figure S6 | Ligand hydrogen bonds interactions.

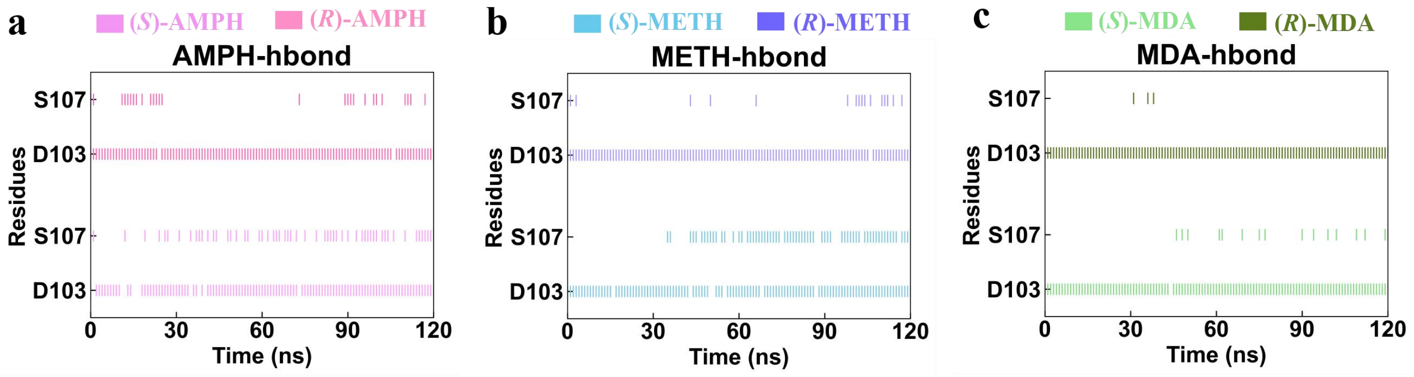

a, b, c The hydrogen bonds interactions between isomers and residues D103$^{3.32}$, S107$^{3.36}$. Pink, *S*-AMPH; Hot pink, *R*-AMPH; Blue, *S*-METH; Purple, *R*-METH; Lime, *S*-MDA; Olive, *R*-MDA.

Figure S7 | Structure determination of METH bound TAAR1–G_s complexes.

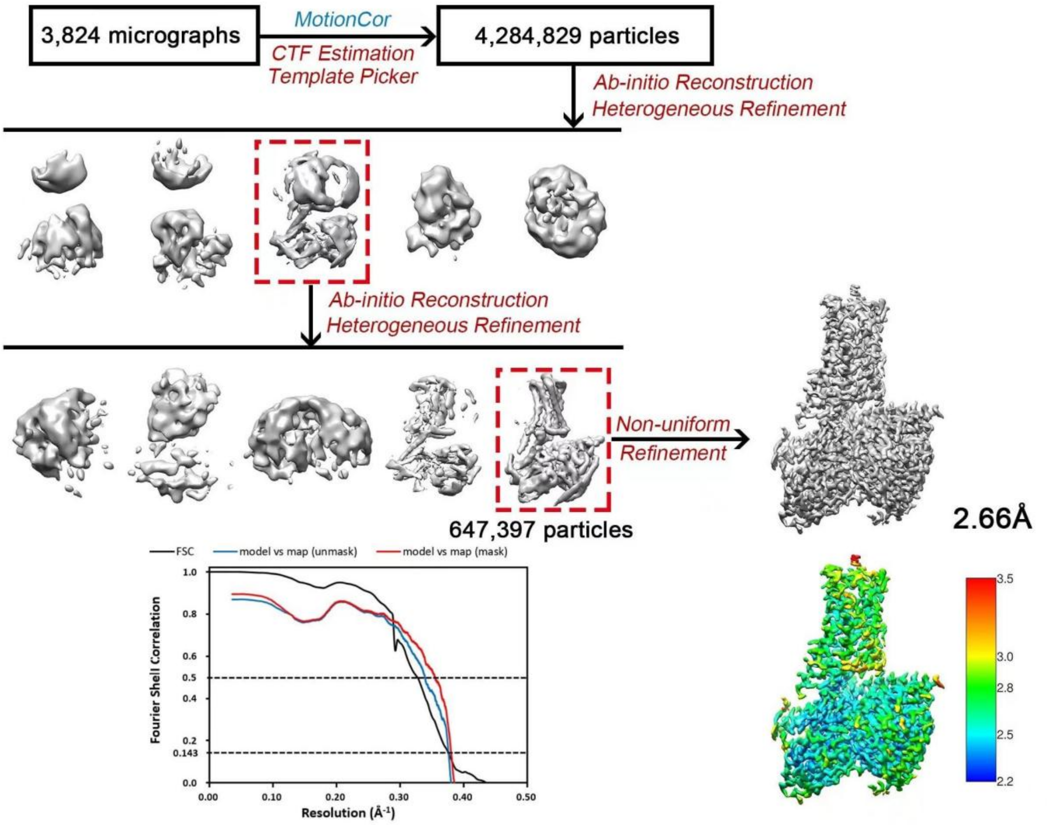

Flow chart of cryo-EM data processing of the METH−TAAR1 complex (PDB ID: 9JKQ). And Gold-standard Fourier shell correlation (GSFSC) curves of the METH−TAAR1 complex.

Figure S8 | Cryo-EM density maps and models of TM1-7, and Helix8 of TAAR1.

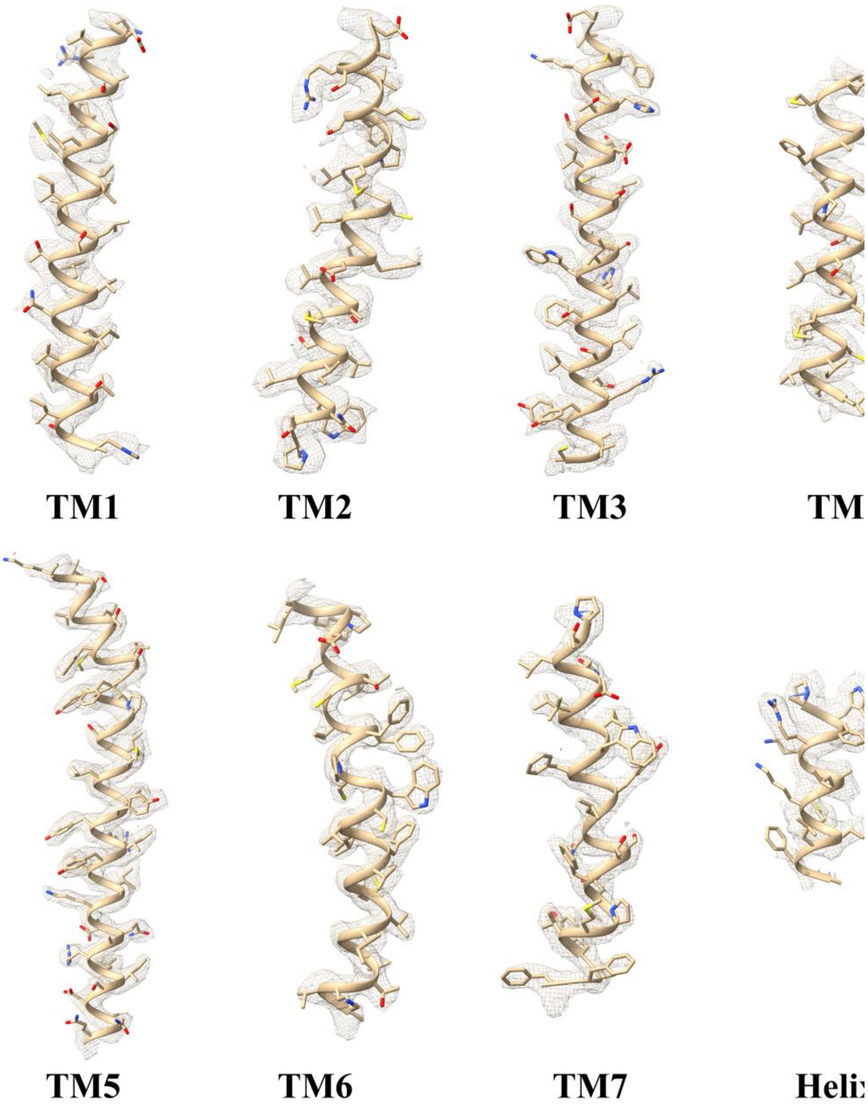

Table S1 | Cryo-EM data collection, model refinement and validation statistics

	**METH−TAAR1−G_s−Nb35**
**Data collection and processing**	
Magnification	105,000
Voltage (kV)	300
Electron exposure ($e^-/Å^2$)	54
Defocus range (μm)	-1.0 ~ -1.5
Pixel size (Å)	0.851
Symmetry imposed	C1
Initial particle projections (no.)	4,284,829
Final particle projections (no.)	647,397
Map resolution (Å)	2.66
FSC threshold	0.143
Map resolution range (Å)	1.85 ~ 39.96
**Refinement**	
Initial model used	XXXX
Model resolution (Å)	2.82
FSC threshold	0.5
Map sharpening B factor ($Å^2$)	-115.4
Model composition	
Non-hydrogen atoms	8,386
Protein residues	1,053
Ligand	1
B-factors ($Å^2$)	
Protein	12.44/141.75/60.52
Ligand	49.97/49.97/49.97
R.m.s. deviations	
Bond lengths (Å)	0.004
Bond angles (°)	0.645
Validation	
MolProbity score	1.40
Clashscore	5.46
Rotamer outliers (%)	0.00
Ramachandran plot	
Favored (%)	97.50
Allowed (%)	2.50
Disallowed (%)	0.00

Table S2 | Engineered TAAR1 construct cAMP signaling assays

TAAR1 Mutants	METH								Expression[e]	
	EC50[a] (μM)	pEC50[b]		Basal[c]		Emax[c]		n[d]		
		mean ±s.e.m.	P value	subtract WT	P value	% of WT	P value		% of WT	P value
WT	5.89	5.23±0.21	/	0	/	100	/	3	100	/
Bril	2.63	5.58±0.17	0.4419	2±1	0.9996	122±6	0.8311	3	140±6*	0.0484
H63$^{2.44}$V	1.70	5.77±0.13	0.0728	5±5	0.9883	137±10	0.3003	3	114±6	0.9087
F112$^{3.41}$W	1.70	5.77±0.03	0.0742	8±0.2	0.8716	196±3***	0.0002	3	103±6	0.9997
c-8	3.63	5.44±0.16	0.8949	0.2±1	>0.9999	125±15	0.7205	3	99±6	>0.9999
F112$^{3.41}$W+Bril	1.95	5.71±0.10	0.1413	27±8**	0.0041	185±14***	0.0008	3	154±10**	0.0038
F112$^{3.41}$W+H63$^{2.44}$V	2.14	5.67±0.12	0.2077	26±7**	0.0065	213±10***	<0.0001	3	104±8	0.9996
F112$^{3.41}$W+c-8	1.82	5.74±0.13	0.1038	6±2	0.9683	203±5***	<0.0001	3	103±8	0.9997
F112$^{3.41}$W+Bril+c-8	1.78	5.75±0.05	0.0865	22±1*	0.0204	216±23***	<0.0001	3	242±11***	<0.0001
F112$^{3.41}$W+Bril+H63$^{2.44}$V	2.14	5.67±0.18	0.1922	42±8***	<0.0001	203±17***	<0.0001	3	269±10***	<0.0001
F112$^{3.41}$W+H63$^{2.44}$V+c-8	1.82	5.74±0.09	0.0954	47±2***	<0.0001	201±9***	0.0001	3	109±13	0.9918
Bril+H63$^{2.44}$V+F112$^{3.41}$W+c-8	4.79	5.32±0.15	0.9994	98±8***	<0.0001	302±19***	<0.0001	3	311±17***	<0.0001

[a]EC_{50} values were determined after 0.5 h stimulation by increasing concentrations of METH at 37°C temperature.
[b]pEC_{50}=-log[EC_{50}].
[c]Emax: the maximal METH response ; Basal: the vehicle (no ligand) response.
[d] Sample size; the number of independent experiments performed in triplicate.
[e] Protein expression levels of TAAR1 constructs at the cell surface were determined in parallel by flowcytometry with an anti-FLAG antibody and reported as percent compared to the WT TAAR1 from at least three independent measurements.
Data are shown as mean±s.e.m. from at least three independent experiments performed in technical triplicate. *P < 0.05, **P < 0.01, ***P < 0.001, one-way ANOVA followed by Dunnett post-test,compared with the response of the WT.

Table S3 | TAAR1 Mutants construct cAMP signaling assays

TAAR1 Mutants	METH						PEA						Expression[e]	
	EC50[a] (μM)	pEC50[b]		Emax[c]		n[d]	EC50[a] (μM)	pEC50[b]		Emax[c]		n[d]	% of WT	P value
		mean ±s.e.m.	P value	% of WT	P value			mean ±s.e.m.	P value	% of WT	P value			
WT	5.86	5.23±0.21	/	100	/	3	2.23	5.65±0.18	/	100	/	3	100	/
D103$^{3.32}$A	nd	nd	nd	nd	nd	3	nd	nd	nd	nd	nd	3	91±5	0.9460
I104 $^{3.33}$A	nd	nd	nd	nd	nd	3	nd	nd	nd	nd	nd	3	91±16	0.9878
S107 $^{3.36}$A	nd	nd	nd	nd	nd	3	nd	nd	nd	nd	nd	3	85±2	0.6338
S108$^{3.37}$A	3.37	4.47±0.04*	0.0242	54±5**	0.0077	3	5.7	5.24±0.13	0.6904	56±8	0.1197	3	98±10	0.9997
V184^{ECL2}A	6.84	5.17±0.18	0.9996	54±6**	0.0073	3	2.48	5.61±0.05	0.9998	116±13	0.9262	3	86±10	0.6906
F186^{ECL2}A	nd	nd	nd	nd	nd	3	nd	nd	nd	nd	nd	3	88±2	0.8813
T194$^{5.42}$A	4.23	5.37±0.15	0.9904	52±2**	0.005	3	0.86	6.06±0.41	0.6788	36±2*	0.0118	3	68±7**	0.0078
F195$^{5.43}$A	0.41	6.39±0.24***	0.0006	186±7***	<0.0001	3	1.49	5.83±0.18	0.9935	156±20*	0.03	3	75±3*	0.0382
F195$^{5.43}$W	nd	nd	nd	nd	nd	3	nd	nd	nd	nd	nd	3	57±7***	0.0001
S198$^{5.46}$A	9.29	5.03±0.18	0.9348	114±2	0.8067	3	5.74	5.24±0.30	0.6825	77±8	0.6851	3	34±2***	<0.0001
F199$^{5.47}$A	nd	nd	nd	nd	nd	3	nd	nd	nd	nd	nd	3	69±3*	0.0108
Y200$^{5.48}$A	0.50	6.30±0.15**	0.0014	283±22***	<0.0001	3	0.45	6.35±0.29	0.1824	187±22***	0.0007	3	96±12	0.9994
W264$^{6.48}$A	nd	nd	nd	nd	nd	3	nd	nd	nd	nd	nd	3	105±7	0.9991
F267$^{6.51}$A	nd	nd	nd	nd	nd	3	nd	nd	nd	nd	nd	3	73±2*	0.0326
F268$^{6.52}$A	1.35	4.89±0.10	0.5109	72±1	0.1516	3	0.79	6.11±0.04	0.5874	104±11	0.9997	3	92±5	0.9893
I290$^{7.39}$A	0.94	6.03±0.14*	0.0174	34±6***	0.0002	3	2.55	5.59±0.12	0.9997	72±9	0.5242	3	79±2	0.1976
G293$^{7.42}$A	nd	nd	nd	nd	nd	3	nd	nd	nd	nd	nd	3	74±5	0.0506
Y294$^{7.43}$A	nd	nd	nd	nd	nd	3	nd	nd	nd	nd	nd	3	63±2**	0.0013

[a]EC_{50} values were determined after 0.5 h stimulation by increasing concentrations of METH at 37°C temperature.
[b]pEC_{50}=-log[EC_{50}].

[c]Emax: the maximal METH response; ND: not detected.

[d] Sample size; the number of independent experiments performed in triplicate.

[e] Protein expression levels of TAAR1 constructs at the cell surface were determined in parallel by flowcytometry with an anti-FLAG antibody and reported as percent compared to the WT TAAR1 from at least three independent measurements.

Data are shown as mean±s.e.m. from at least three independent experiments performed in technical triplicate. *$P < 0.05$, **$P < 0.01$, ***$P < 0.001$, one-way ANOVA followed by Dunnett post-test,compared with the response of the WT.

Table S4 | The binding free energies of the *S*- and *R*-configurations of AMPH/ METH/ MDA

Amphetamine-like ligands		*S* type		*R* type		P vaule
		energy	n	energy	n	
	METH	-19.56±0.53	30	-17.84±0.48*	30	0.0354
**Total**	AMPH	-18.08±0.32	30	-15.57±0.61***	30	0.0009
	MDA	-19.48±0.47	30	-18.15±0.41	30	0.1556
	METH	-1.90±0.47	30	0.48±0.56**	30	0.0100
**S107**	AMPH	-1.74±0.48	30	0.31±0.13*	30	0.0329
	MDA	-4.32±0.93	30	-0.57±0.51***	30	<0.0001

Data are shown as mean±s.e.m. from at least thirty independent experiments. $*P < 0.05$, $**P < 0.01$, $***P < 0.001$, two-way ANOVA followed by Bonferroni test, compared with *S* type.
